# Supplementary material for: Dynamics of HIV Reservoir and HIV-1 Viral Splicing in HCV-Exposed Individuals after Elimination with DAAs or Spontaneous Clearance
Source: J Clin Med. 2022 Jun 21;11(13):3579. doi: 10.3390/jcm11133579 (PMC9267476; doi:10.3390/jcm11133579)
Supplement: Supplementary file 1 [file jcm-11-03579-s001.zip › File S1.pdf]

File S1. COVIHEP and Methods

Study design
Long-term prospective cohort study.

Setting
Health centres
Three tertiary Hospitals of the Spanish National Health System belonging to the Group of COinfection on HIV and viral Hepatitis (COVIHEP).

COVIHEP
It is a multidisciplinary research group where virologists, clinicians, immunologists, biochemists, biologists, bioinformaticians and statisticians work together and in a coordinated manner to achieve a better understanding of the prognosis of HIV infection in patients exposed to chronic viral hepatitis.

Multidisciplinary group of HIV/Hepatitis viral coinfection (COVIHEP) [In alphabetical order of institutions and authors within each institution]:

- **Hospital Universitario 12 de octubre (Madrid-Spain)**: Laura Bermejo-Plaza; Otilia Bisbal; Lourdes Domínguez-Domínguez; María Lagarde; Mariano Matarranz; Federico Pulido; Rafa Rubio; Mireia Santacreu.
- **Hospital Universitario Infanta Leonor (Madrid-Spain)**: Guillermo Cuevas; Victorino Diez-Viñas; Pablo Ryan; Jesús Troya.
- **Hospital Universitario La Paz (Madrid-Spain)**: Juan Miguel Castro-Álvarez; Marta Gálvez-Charro; Luz Martín-Carbonero; Mario Mayoral-Muñoz.
- **Hospital Universitario La Princesa (Madrid-Spain)**: Ignacio de los Santos; Lucio García-Fraile; Jesús Sanz-Sanz.
- **Hospital Universitario Puerta del Hierro Majadahonda (Madrid-Spain)**: Alfonso Ángel-Moreno; Sara de la Fuente Moral.
- **Instituto de salud Carlos III Majadahonda (Madrid-Spain)**: José Alcamí; Verónica Briz, Oscar Brochado; Maria Teresa Coiras; Alicia Gómez-Sanz, Amanda Fernández-Rodríguez; Paula Martínez-Román; Salvado Resino; Marta Sánchez-Carrillo
- **Research Institute for Medicines (Lisboa-Portugal)**: Claudia Palladino; Nuno Taveira
- **The Roslin Institute (Edinburgh-UK) and CENIDCI, Centro Nacional de Investigación y Desarrollo del Cerdo Ibérico of INIA (Madrid-Spain)**: María Muñoz-Muñoz.

Location
The Madrid region, *Comunidad de Madrid* (Spain).

Cohort
Spanish Cohort of HIV/HCV coinfected individuals that belongs to the COVIHEP group.
This cohort is formed by HIV+ patients previously exposed to HCV (N=220) who have eliminated the HCV spontaneously or with DAAs (n=120). The cohort was created in 2015 after the adjudication of the national competitive project PI15CIII/00031, in which were enrolled samples from **220 HIV+ patients exposed to HCV** of whom some of them (N=80) initiated HCV treatment with DAAs. In 2018, the adjudication of the project PI18/00020, allowed to incorporate **120 additional samples 48 weeks after HCV elimination, spontaneously or by DAAs.** This second sample was collected 48 weeks after reach HCV

sustained virological response. In HIV+ and HIV+/HCV- individuals baseline and end-point samples differ also 48 weeks. Samples are stored in the ISCIII Biobank (collection C.0003822). Clinical and epidemiological data from both infections, including more than 30 variables, are also collected in an electronic data capture tool (RedCap).

Study period

Initiation date

Recruitment: 2016-2017

End date

Recruitment: 2018-2019

Participants' selection

Participants have been recruited from five tertiary hospitals from the Madrid region (Spain), who had been enrolled in the parental study "López-Huertas MR, Palladino C, Garrido-Arquero M, Esteban-Cartelle B, Sánchez-Carrillo M, Martínez-Román P, et al. HCV-coinfection is related to an increased HIV-1 reservoir size in cART-treated HIV patients: a cross-sectional study. Sci Rep. 2019;9(1):5606" [19].

Inclusion criteria

People leaving with HIV (PLWH), with age more than 18 years old and on suppressive cART who had been undetectable for HIV during the previous year before enrolment and had CD4+ T-cells ≥500 cells/mm^3 since at least one year before sample collection.

Exclusion criteria

Hepatic decompensation, alcohol-related liver damage, presence of viral hepatitis B antigens or antibodies against viral hepatitis B, opportunistic infections, drug abuse and addiction, other diseases (diabetes, nephropathies, autoimmune diseases, hemochromatosis, cryoglobulinemia, primary biliary cirrhosis, Wilson's disease, deficiency of alpha1 antitrypsin and neoplasia), pregnancy.

Study groups

Subjects were included in one of the three following groups according to their HCV status:
1) HIV+ monoinfected group: PLWH who were negative for both HCV PCR and antibodies;
2) HIV+/HCV- group: PLWH coinfected with HCV who had spontaneously cleared HCV infection (negative HCV PCR but positive HCV antibodies) during the first 6 months after HCV infection;
3) HIV+/HCV+ group: PLWH chronically infected HCV (positive HCV PCR and antibodies) naïve to any HCV treatment at baseline, but who eliminated the hepatic virus (achieved SVR) with DAAs at endpoint.

Methods of follow-up

Enrollment was carried out between November 2016 and June 2017 for all study groups. Subsequently, medical visits were carried out every 3 months until the end of follow-up, when SVR was confirmed. Overall, the follow up ended between January 2018 and March 2019. After 48 weeks from SVR confirmation, the endpoint sample was collected for each patients. Overall, the follow-up was of at least 60 weeks.

Biological samples

All baseline blood samples were collected at enrollment, before DAAs initiation. and endpoint samples from January 2018 to March 2019

Peripheral blood mononuclear cells (PBMCs) and plasma. 60 ml of blood (in EDTA) were drawn from each patient due to the need to isolate resting (r) CD4 T-cells.

Variables:

Clinical and epidemiological data, weight, BMI, smoking habits, metabolic markers of lipid profile and liver functions, etc, were recorded from medical records. For HCV: fibrosis stage change, necroinflammatory activation and hepatic steatosis variation. For HIV: viral load, CD4/CD8 ratio and CD4+ nadir.

For the purpose of the present study, those variables were also recorded: DAAs, HCV genotype.

HIV infection diagnosis

Plasma HIV-1 RNA viral load was measured by Amplicor Monitor assay (Roche Diagnostic Systems Inc., Branchburg, New Jersey, USA) and real-time NASBA (Easy Mag y Nuclisens Easy Q; BioMerieux, Marcy l'Etoile, France) with a detection limit of 20 copies/ml (undetectable viral load)

HCV infection diagnosis

Plasma HCV RNA viral load was measured by COBAS® TaqMan® HCV Test v2.0 (Roche Diagnostic Systems Inc., Branchburg, New Jersey, USA).

Liver fibrosis was performed by transient elastography (TE) (FibroScan-502® (Echosens, Paris) using the following cut-off values for fibrosis of kPa to identify the different stages of fibrosis: F0-F1 (<6kPA), F2 (6-9kPa), F3 (>9-12kPa) and F4 (>12kPa)

IFNL3 rs12979860 single nucleotide polymorphism analysis was performed by qPCR using a custom taqman polymorphism assay (Life Technologies, California, USA) for rs12979860.

cART

All patients presented a Fiebig stage ≥V when initiated cART.

Thirty-seven percent of the patients were receiving a cART regimen based on integrase inhibitors and 31% on NNRTIs. More than 20% of patients were receiving dual or monotherapy based on protease inhibitors.

Adherence

Pharmacy refill records and individuals interviews.

Blips

A blip was defined as any HIV RNA of 20–999 copies/mL immediately preceded and followed by HIV RNA of less than 20 copies/mL without a change in ART.

DAAs

Information related to the different HCV treatments with DAAs is shown in Table 3: Clinical characteristics of the study population related to HCV-infection.

SVR definition

Sustained virological response (SVR), determined in an on-treatment approach and defined as undetectable HCV RNA 12 weeks after the scheduled end of therapy (SVR_{12}).

Data sources/ measurement

The epidemiological and clinical variables of study participants were collected from medical records. All methods were rigorously applied consistently to the three study groups included, allowing for comparability of results

Routine laboratory measures

Plasma HIV-1 RNA was measured using the commercial quantitative Amplicor Monitor assay (Roche diagnostic Systems, USA) with a detection limit of 50 copies/mL every 3 months. For the purpose of the present study, baseline and endpoint viral load are presented.

$CD4^{+}$ T-lymphocytes were quantified by flow cytometry (Coulter, Madrid, Spain) every 3 months. For the purpose of the present study, baseline and endpoint viral loads are presented.

Study size

Power/sample size: accepting an alpha risk of 0.05 and a beta risk of 0.2 in a two-sided test, the result is 30 patients per group, with a correlation coefficient of 0.5. A dropout rate of 0% is expected.
