# Supplementary material for: Dynamics of HIV Reservoir and HIV-1 Viral Splicing in HCV-Exposed Individuals after Elimination with DAAs or Spontaneous Clearance
Source: J Clin Med. 2022 Jun 21;11(13):3579. doi: 10.3390/jcm11133579 (PMC9267476; doi:10.3390/jcm11133579)
Supplement: Supplementary file 1 [file jcm-11-03579-s001.zip › File S2.pdf]

File S2: HIV viral reservoir size (proviral HIV DNA copies/10^6 cells) of the three different study groups at baseline and endpoint in resting CD4+ T cells (rCD4+ T cells)

**rCD4+ T cells**					
**BASELINE**					
**Groups**	**Mean ± SEM**	**AMR (95% CI)**	***p***	**aAMR (95% CI)**	***p***
**HIV+**	279.54 ± 69.00	0	-	0	-
**HIV+/HCV-**	1276.22 ± 419.46	4.565 (1.919; 10,911)	***0.001***	4.146 (1.611; 10.669)	***0.003***
**HIV+/HCV+**	680.29 ± 207.82	2.434 (1.097; 5.396)	***0.029***	2.878 (1.221; 6.784)	***0.016***
**ENDPOINT**					
**Groups**	**Mean ± SEM**	**AMR (95% CI)**	***p***	**aAMR (95% CI)**	***p***
**HIV+**	956.51 ± 74.00	0	-	0	-
**HIV+/HCV-**	852.06 ± 232.06	0.891 (0.330; 2.407)	0.820	2.093 (0.759; 5.772)	0.154
**HIV+/HCV+**	2398.81 ± 1572.16	2.508 (1.011; 6.222)	***0.047***	4.679 (1.846; 11.859)	***0.001***

Note: HIV, Human Immunodeficiency Virus; HCV, Hepatitis C Virus; rCD4+ T cells, resting CD4+ T cells; rCD4 T- PBMCs, resting CD4 T cells-depleted PBMCs; AMR, arithmetic mean ratio from univariate analysis; aAMR, adjusted arithmetic mean ratio from multivariate analysis; SEM, standard error of the mean; baseline, time of the study when HIV+/HCV+ individuals had never been treated for hepatitis; endpoint, time of the study when HIV+/HCV+ subjects had cleared HCV by treatment with direct-acting antivirals. Both the univariate GLM and the multivariate GLM were used to calculate differences between the HIV+ control group and the HIV+/HCV- and HIV+/HCV+ groups. Statistical significance was defined as $P < 0.05$ (2-tailed).

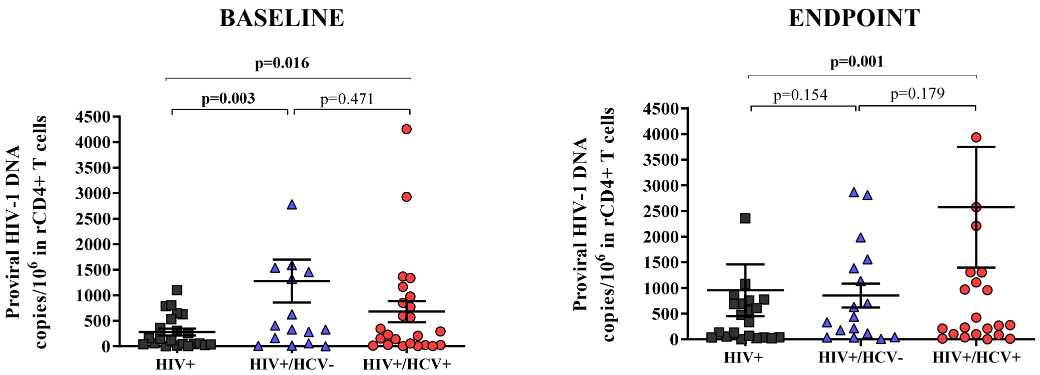

Note: HIV, Human Immunodeficiency Virus; HCV, Hepatitis C Virus; rCD4+ T cells, resting CD4+ T cells; rCD4 T- PBMCs. resting CD4 T cells-depleted PBMCs; baseline, time of the study when HIV+/HCV+ patients had never been treated for hepatitis; endpoint, time of the study when HIV+/HCV+ patients had cleared HCV by treatment with direct-acting antivirals. Dot plot error bars represent reservoir size arithmetic mean and standard error of the mean. Gamma-distributed multivariate GLM adjusted by time of infection, total CD4+T cells and HIV cART was used to calculate differences between the HIV+ control group and the HIV+/HCV- and HIV+/HCV+ groups. Statistical significance was defined as $P < 0.05$ (2-tailed).
