# Supplementary material for: Dynamics of HIV Reservoir and HIV-1 Viral Splicing in HCV-Exposed Individuals after Elimination with DAAs or Spontaneous Clearance
Source: J Clin Med. 2022 Jun 21;11(13):3579. doi: 10.3390/jcm11133579 (PMC9267476; doi:10.3390/jcm11133579)
Supplement: Supplementary file 1 [file jcm-11-03579-s001.zip › File S3.pdf]

File S3: HIV viral reservoir size (proviral HIV DNA copies/10^6 cells) of the three different study groups at baseline and endpoint in resting CD4 T cells-depleted PBMCs (rCD4 T- PBMCs)

**rCD4 T- PBMCs**					
**BASELINE**					
**Groups**	**Mean ± SEM**	**AMMR (95% CI)**	***p***	**aAMR (95% CI)**	***p***
**HIV+**	249.45 ± 90.85	0	-	0	-
**HIV+/HCV-**	599.04 ± 268.34	11.079 (0.318; 385.554)	0.184	2.442 (0.840; 7.100)	0.101
**HIV+/HCV+**	828.73 ± 723.49	7.512 (3.27; 96.023)	0.704	1.469 (0.389; 5.553)	0.571
**ENDPOINT**					
**Groups**	**Mean ± SEM**	**AMR (95% CI)**	***p***	**aAMR (95% CI)**	***p***
**HIV+**	898.34 ± 273.23	0	-	0	-
**HIV+/HCV-**	873.65 ± 397.18	0.973 (0.374; 2.528)	0.954	1.329 (0.474; 3.726)	0.589
**HIV+/HCV+**	1035.28 ± 467.13	1.152 (0.461; 2.879)	0.761	1.063 (0.378; 2.995)	0.907

Note: HIV, Human Immunodeficiency Virus; HCV, Hepatitis C Virus; rCD4+ T cells, resting CD4+ T cells; rCD4 T- PBMCs, resting CD4 T cells-depleted PBMCs; AMR, arithmetic mean ratio from univariate analysis; aAMR, adjusted arithmetic mean ratio from multivariate analysis; SEM, standard error of the mean; baseline, time of the study when HIV+/HCV+ individuals had never been treated for hepatitis; endpoint, time of the study when HIV+/HCV+ subjects had cleared HCV by treatment with direct-acting antivirals. Both the univariate GLM and the multivariate GLM were used to calculate differences between the HIV+ control group and the HIV+/HCV- and HIV+/HCV+ groups. Statistical significance was defined as $P < 0.05$ (2-tailed).

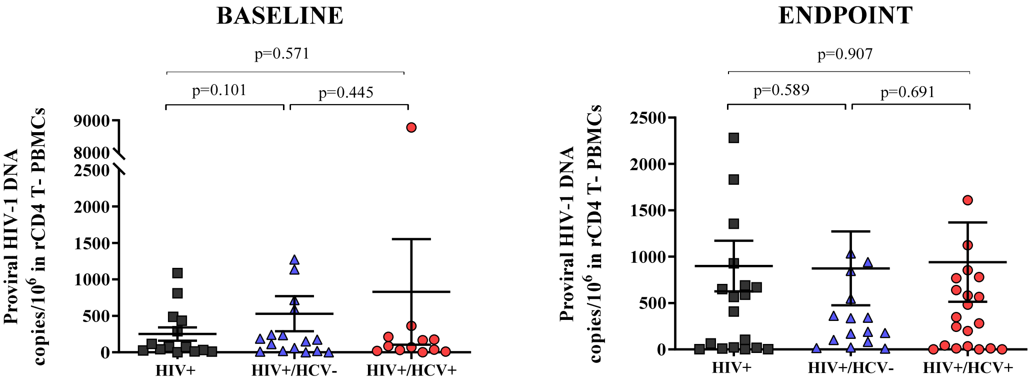

Note: HIV, Human Immunodeficiency Virus; HCV, Hepatitis C Virus; rCD4+ T cells, resting CD4+ T cells; rCD4 T- PBMCs, resting CD4 T cells-depleted PBMCs; baseline, time of the study when HIV+/HCV+ patients had never been treated for hepatitis; endpoint, time of the study when HIV+/HCV+ patients had cleared HCV by treatment with direct-acting antivirals. Dot plot error bars represent reservoir size arithmetic mean and standard error of the mean. Gamma-distributed multivariate GLM adjusted by time of infection, total CD4+T cells and HIV cART was used to calculate differences between the HIV+ control group and the HIV+/HCV- and HIV+/HCV+ groups. Statistical significance was defined as $P < 0.05$ (2-tailed).
