# Supplementary material for: Dynamics of HIV Reservoir and HIV-1 Viral Splicing in HCV-Exposed Individuals after Elimination with DAAs or Spontaneous Clearance
Source: J Clin Med. 2022 Jun 21;11(13):3579. doi: 10.3390/jcm11133579 (PMC9267476; doi:10.3390/jcm11133579)
Supplement: Supplementary file 1 [file jcm-11-03579-s001.zip › File S4.pdf]

File S4: HIV viral splicing (ΔRQ) of the three different study groups at baseline and endpoint in resting CD4+ T cells (rCD4+ T cells)

**rCD4+ T cells**						
**BASELINE**						
**Groups**	**Unspliced Mean ± SEM**	***p***	**Single spliced Mean ± SEM**	***p***	**Multiple spliced Mean ± SEM**	***p***
**HIV+**	76.51 ± 20.46	0.601	52.47 ± 47.92	0.576	3.06 ± 3.06	0.489
**HIV+/HCV-**	211.66 ± 87.79		53.68 ± 27.28		0 ± 0	
**HIV+/HCV+**	60.54 ± 17.69		298.00 ± 219.14		0.05 ± 0.04	
**ENDPOINT**						
**Groups**	**Unspliced Mean ± SEM**	***p***	**Single spliced Mean ± SEM**	***p***	**Multiple spliced Mean ± SEM**	***p***
**HIV+**	19427.74 ± 10636.42	0.054	5248.19 ± 1930.61	0.121	45.29 ± 43.01	0.956
**HIV+/HCV-**	25931.78 ± 9188.52		2937.77 ± 1164.09		159.02 ± 158.98	
**HIV+/HCV+**	6720.26 ± 1730.39		915.50 ± 508.29		43.80 ± 39.3	

Note: HIV Human Immunodeficiency Virus; HCV, Hepatitis C Virus; rCD4+ T cells, resting CD4+ T cells; rCD4 T- PBMCs, resting CD4 T cells-depleted PBMCs; SEM, standard error of the mean. A Kruskall-Wallis H-test was used to compare differences between study groups for the different forms of splicing. Statistical significance was defined as $P < 0.05$ (2-tailed).

BASELINE rCD4+ T cells

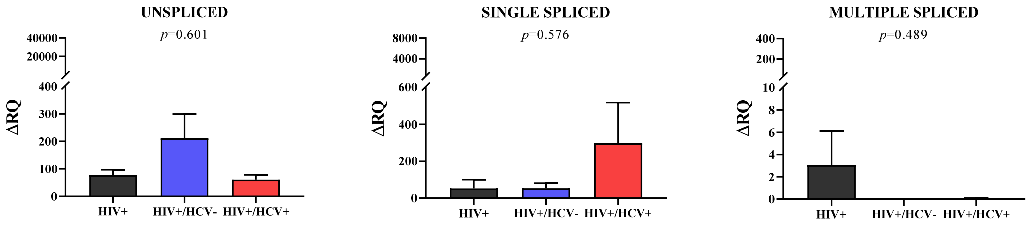

ENDPOINT rCD4+ T cells

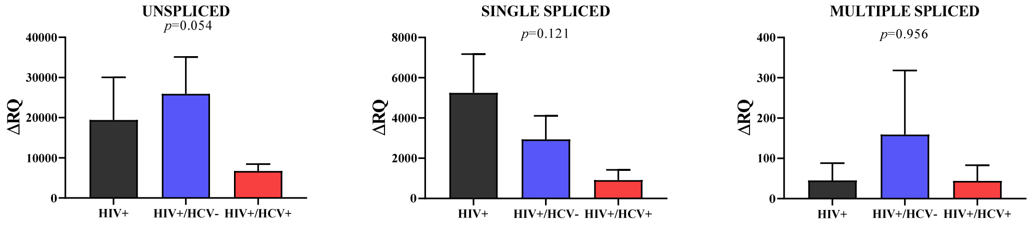

Note: HIV, Human Immunodeficiency Virus; HCV, Hepatitis C Virus; rCD4+ T cells, resting CD4+ T cells; rCD4 T- PBMCs, resting CD4 T cells-depleted PBMCs; baseline, time of the study when HIV+/HCV+ individuals had never been treated for hepatitis; endpoint, time of the study when HIV+/HCV+ subjects had cleared HCV by treatment with direct-acting antivirals. Bars represent ΔRQ arithmetic mean and standard error of the mean. A Krusall-Wallis H-test was used to determine whether the three study groups behaved as independent populations. Statistical significance was defined as $P < 0.05$ (2-tailed).
