# Supplementary material for: Dynamics of HIV Reservoir and HIV-1 Viral Splicing in HCV-Exposed Individuals after Elimination with DAAs or Spontaneous Clearance
Source: J Clin Med. 2022 Jun 21;11(13):3579. doi: 10.3390/jcm11133579 (PMC9267476; doi:10.3390/jcm11133579)
Supplement: Supplementary file 1 [file jcm-11-03579-s001.zip › File S5.pdf]

File S5: HIV viral splicing (ΔRQ) of the three different study groups at baseline and endpoint in resting CD4 T cells-depleted PBMCs (rCD4 T- PBMCs)

**rCD4 T- PBMCs**						
**BASELINE**						
**Groups**	**Unspliced Mean ± SEM**	***p***	**Single spliced Mean ± SEM**	***p***	**Multiple spliced Mean ± SEM**	***p***
**HIV+**	30.87 ± 11.54	***0.013***	63.25 ± 44.69	0.841	2.80 ± 2.80	0.692
**HIV+/HCV-**	979.10 ± 343.61		1282.70 ± 838.93		0 ± 0	
**HIV+/HCV+**	2784.43 ± 2473.01		2869.30 ± 2414.90		0.03 ± 0.02	
**ENDPOINT**						
**Groups**	**Unspliced Mean ± SEM**	***p***	**Single spliced Mean ± SEM**	***p***	**Multiple spliced Mean ± SEM**	***p***
**HIV+**	17908.59 ± 11459.20	0.310	2645.84 ± 1774.06	0.332	509.98 ± 509.98	0.956
**HIV+/HCV-**	52861.64 ± 22163.51		315926.84 ± 1774.06		6.80 ± 6.80	
**HIV+/HCV+**	106632.50 ± 74472.90		75566.19 ± 52720.25		518.14 ± 512.64	

Note: HIV, Human Immunodeficiency Virus; HCV, Hepatitis C Virus; rCD4+ T cells, resting CD4+ T cells; rCD4 T- PBMCs, resting CD4 T cells-depleted PBMCs; SEM, standard error of the mean. A Kruskall-Wallis H-test was used to compare differences between study groups for the different forms of splicing. Statistical significance was defined as $P < 0.05$ (2-tailed).

BASELINE rCD4 T- PBMCs

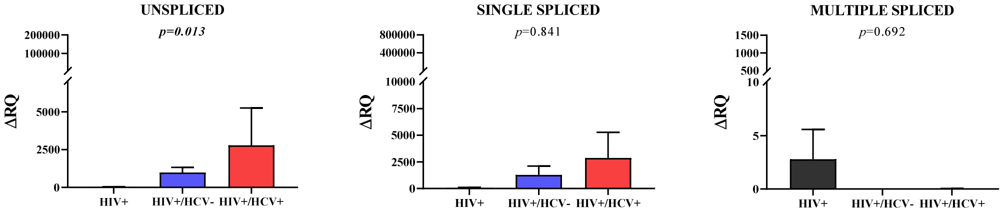

ENDPOINT rCD4 T- PBMCs

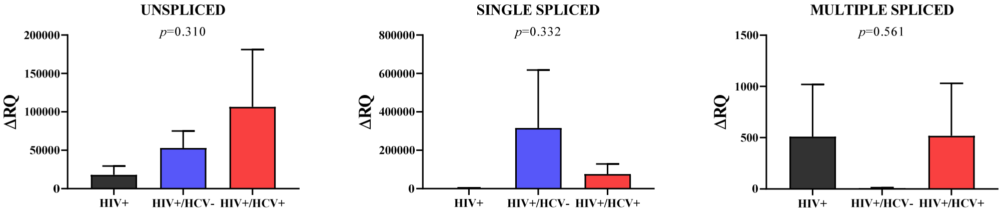

Note: HIV, Human Immunodeficiency Virus; HCV, Hepatitis C Virus; rCD4+ T cells, resting CD4+ T cells; rCD4 T- PBMCs, resting CD4 T cells-depleted PBMCs; baseline, time of the study when HIV+/HCV+ individuals had never been treated for hepatitis; endpoint, time of the study when HIV+/HCV+ subjects had cleared HCV by treatment with direct-acting antivirals. Bars represent ΔRQ arithmetic mean and standard error of the mean. A Krusall-Wallis H-test was used to determine whether the three study groups behaved as independent populations. Statistical significance was defined as $P < 0.05$ (2-tailed).
